# Application of temporarily functional antibiotic-containing bone cement prosthesis in revision hip arthroplasty

**DOI:** 10.1007/s00590-012-1140-7

**Published:** 2012-11-23

**Authors:** Ke Liu, Jia Zheng, Yi Jin, Yong-qiang Zhao

**Affiliations:** Department of Orthopedics, Henan Provincial People’s Hospital, No. 7, Weiwu Road, 450003 Zhengzhou, China

**Keywords:** Infection, Antibiotic-impregnated cement prosthesis, Revision total hip arthroplasty

## Abstract

**Purpose:**

To investigate the clinical outcome of two-stage revision total hip arthroplasty for infected hip arthroplasty using antibiotic-impregnated cement prosthesis.

**Materials and methods:**

Forty-one patients, who suffered from an infection after hip replacement or internal fixation of femoral neck and trochanteric fractures, were treated with a two-stage revision hip arthroplasty and followed up for an average of 37 months. All the patients were implanted with antibiotic-impregnated cement prosthesis as one-stage treatment and were then managed with two-stage revision hip arthroplasty after 12–24 weeks. During the follow-up, Merle d’Aubigné hip score and Harris score were employed for assessment of hip function, and infection recurrence was observed.

**Results:**

According to Merle d’Aubigné hip score, 16 patients (39.2 %) were excellent, 19 (46.3 %) were good, 6 (14.6 %) were moderate, and no bad result and the average score was 15.42. Mean Harris score of preoperation, interval period, and postoperation was 46.7, 66.5, and 92.3, respectively. There was no infection recurrence.

**Conclusion:**

Two-stage revision total hip arthroplasty for infected hip arthroplasty using antibiotic-impregnated cement prosthesis has a satisfying clinical outcome.

## Introduction

Hip infections include post-total hip replacement (THR) infection, infection after internal fixation for femoral neck and intertrochanteric fractures, and primary infection [1]. Postoperative infection is the most severe post-THR complication. Although the incidence of postoperative infection only ranges from 1 to 2 % [2], its disastrous consequences do not only impose great pains and high medical costs on patients, but become the difficulties as well as the bottle neck in joint surgery.

Nowadays, surgical treatment methods for infected hips mainly include debridement and prosthetic retention, one-stage revision hip arthroplasty (the infected joint prosthesis is taken out, and a new one is then implanted in one operation), and two-stage revision hip arthroplasty (the first operation is performed for foreign body removal and thorough debridement, and the second is performed for new prosthesis implantation after some time of infection healing). For patients with obstinate repeated infection or infection which poses life threat, hip fusion or amputation may even be performed. With the successful application of prostalac system in joint infection treatment, two-stage revision hip arthroplasty has become the gold standard in the treatment of post-THR infection. Jackson and Schmalzried [3] analyzed retrospectively the reports on one-stage revision hip arthroplasty before 2,000 and discovered that 1,077 of 1,229 hip infections were well controlled with a success rate of 82.9 %. Hanssen and Spangehl [4] reported that the infection control rate of two-stage revision hip arthroplasty reached 90–100 %. Garvin and Hanssen [5] reported that the success rate of one-stage revision hip arthroplasty for 1,189 patients with postoperative infection was 82 %, whereas that of two-stage revision for 423 patients reached 91 %. Two-stage revision hip arthroplasty has an obviously better effect on post-THR infection than one-stage revision. In two-stage revision, the key steps lie in thorough debridement, and the application of cement spacer mixed with antibiotics. The temporary prosthesis can create a local highly concentrated antibiotic environment during the interval between the first and second surgical procedures to effectively kill bacteria. This ensures that the infection is well controlled on the one hand and that the revision success rate is increased on the other. Further, the temporary prosthesis can keep the tension of soft tissues intact. Partial body weight-bearing walking allowed during the interval between the first and second operations can help patients maintain their affected hip function. Even though, Wentworth et al. [6] reported that an infection recurrence rate between 10 and 15.1 % still occurs after one- or two-stage revision hip arthroplasty involving the application of antibiotic-containing bone cement. Therefore, there are still many questions pertaining to the treatment of infected hips to be solved.

The authors of the current study conducted long-term explorations into how to increase the clinical recovery rate of infected hips and improve patients’ hip function and life quality and how to standardize the whole treatment procedure. To achieve these goals, a temporarily functional patent prosthetic die was developed for prosthetic creation using vancomycin (4 g)—containing bone cement (80 g) which embraced a rigid structure of Steinmann pin and steel wires. At the first stage, the infected prosthesis was taken out, and a temporarily functional prosthesis was implanted after thorough debridement. All patients were allowed to walk with the aid of crutches from 4 weeks after operation. At 12–24 weeks, when infection was completely controlled, they were subjected to the second-stage revision hip arthroplasty. The involved 41 patients (41 hips) were followed up for 37 months. Good results were obtained in infection recovery rate and hip functional rehabilitation.

## Materials and methods

### Clinical data

This study was conducted in accordance with the Declaration of Helsinki. This study was conducted with approval from the Ethics Committee of Henan Provincial People’s Hospital. Written informed consent was obtained from all participants. Between March 2006 and June 2011, we performed two-stage revision hip arthroplasty for a total of 41 patients with infected hip (41 hips). All of them had Tsukayama type IV and late infection. The infected signs were found from 3 months to 2 years after initial THA. Among the patients, 28 were males and 13 were females with an average age of 63.6 years (ranging from 51 to 76 years). All patients suffered from unilateral infection, in which 24 were on the right side and 17 on the left side. Their mean follow-up time was 37 months (ranging from 29 to 61 months) after revision.

**Fig. 1:**
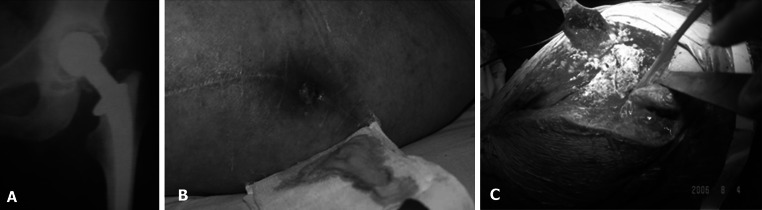
**a** X-Ray films of THR postoperative infection. **b** Sinus formation postoperative infection. **c** Pub substances were observed during operation

Among the 41 infected hips, 2 occurred after dynamic hip screw (DHS) internal fixation for femoral neck fracture, 1 occurred after compression hollow screw internal fixation for femoral neck fracture, 1 occurred after proximal femoral nail (PFN) internal fixation for femoral intertrochanteric fracture, and 34 occurred after THR. Seventeen had sinus formation, 39 had hip persistent pain with continuously increased erythrocyte sedimentation rate (>40 mm/h) and C-reactive protein (>20 mg/L) as well as increased white blood cell count and classification, 6 had positive pre- or intra-operative secretions, and 12 had radiolucent zones (>2 mm) around the prosthesis according to imaging examination. Bone nuclide scanning showed that all the infected hips displayed nuclide accumulations [7]; all the hips were observed with obvious periosteal reactions caused by infection: pub substances, inflammatory granulation tissues, and reactive hyperosteogeny or worm-bitten-like bone defects; and all frozen tissue sections presented a white blood cell count of more than 10 (×400; 10 visual fields) [8] (Fig. 1).

### Operative procedures

**Fig. 2:**
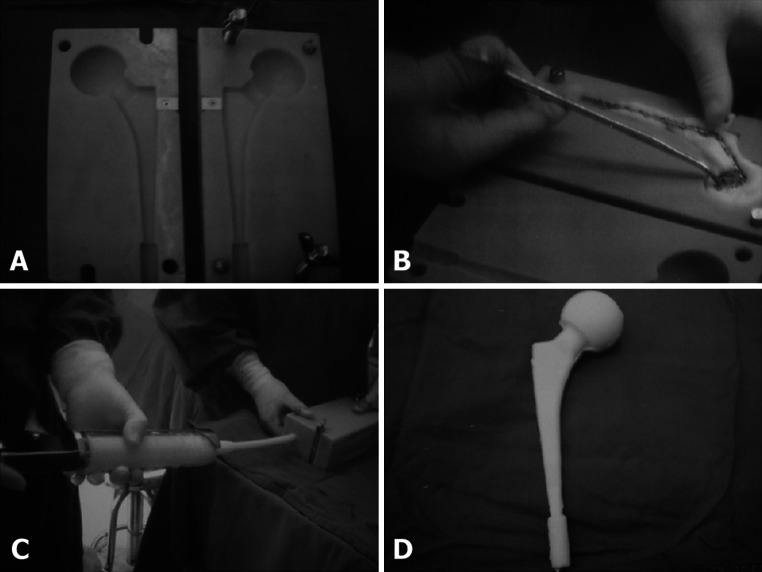
**a** Standard temporary prosthesis mold designed by double-sided grooves gusset plate. **b** Steel skeleton implanted. **c** Pressure injection molding by bone cement containing 5 % vancomycin. **d** Antibiotic bone cement temporary functional prosthesis

**Fig. 3:**
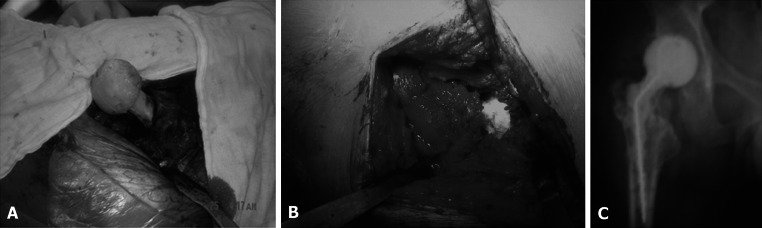
**a** Insertion prosthesis into femoral medullary canal. **b** Restoration of Antibiotic bone cement temporary functional prosthesis. **c** X-ray after implantation

At the first stage, the infected prosthesis was dislocated and taken out. Afterward, the false membranes between the prosthesis and the bone surfaces [9], sinuses, inflammatory granulation tissues, and bone cement were thoroughly removed (particularly the bone cement, for that its remnant is one of the primary causes for treatment failure [4]). Pressurized hydrogen peroxide, metronidazole, and physiologic saline solution irrigations were performed. Surface sequestra were removed using an intramedullary broacher and an acetabular reamer. For hip infection, after internal fixation for femoral proximal fracture, femoral proximal osteotomy used in THR was adopted after internal fixation removal to take out the femoral head and neck, and then, intramedullary broaching was performed but without socket reaming (only infected tissues were cleared there). The bone bed was washed using a sterilized brush and pressed physiologic saline (no less than 3,000 ml). Then, a self-developed standard temporary hip prosthetic die with a fluted clamp on both sides was selected according to the diameter of the femoral head (dies were divided into 44, 48, and 52 mm types according to different diameters) (Fig. 2a). A rigid temporary prosthetic bone framework of a Steinmann pin (2.5 mm in diameter) wound by steel wires was placed in the die (Fig. 2b). Antibiotic-containing bone cement curing liquid at 80 g (containing 4 g of vancomycin) was stirred into wiredrawing and poured into the die (Fig. 2c). After solidification, the created temporary prosthesis was taken out (Fig. 2d). The prosthesis was inserted into the femoral marrow cavity according to the THR position, its anteversion angle was adjusted, and its head was placed into the acetabulum (Fig. 3). The operative region was rinsed with physiologic saline, a drainage tube was detained, and interrupted suturing was then performed layer by layer. For hip infection after internal fixation for femoral proximal fracture, the integrity of hip muscles (especially those important dynamic muscles like the middle gluteal muscle) was protected as much as possible since no previous excision had damaged them. The drainage tube was extracted at 48 h after operation, antibiotics sensitive to bacteria were normally administrated for 6 weeks (to those from which no bacteria were cultured, intravenous administration of vancomycin (vancocin CP) at 0.5 g was given twice a day), and then rifampicin was orally administered for another 6 weeks [10]. Patients were told to take partial body weight-bearing walks four times a day from 4 weeks after operation, with each for approximately 50 m. The walking mainly focused on hip muscular training (the training of the middle gluteal muscle in particular).

The second operation was performed at 12–24 weeks when ESR decreased below 20 mm/h, CRP decreased below 20 mg/L [11], the wound healed well, and the patient had no pain in partial body weight-bearing condition. The operation was performed at the original incision site. Fresh granulation tissues, clear cicatricial tissues, and middle gluteal muscle in good elasticity were observed around the temporary prosthesis. The prosthesis was taken out, the surrounding granulation tissues were removed, bone grafting was performed according to bone bed defects, and a non-cemented total hip prosthesis was then implanted.

## Results

All the 41 infected hips healed with a recovery rate of 100 %. At 12 weeks after the first operation, the mean hip functional score assessed by Merle d’Aubigné’s method was 13.2 with excellent, good, fair, and poor rates of 0, 41.1, 58.9, and 0 %, respectively. The mean Harris score increased to 66.5 from preoperative 46.7. During the interval between the first- and second-operative stages, patients could walk in body weight-bearing condition with the aid of crutches, and their life quality was greatly improved. All the patients were followed up after the second operation with an average follow-up time of 37 months. At the end of follow-ups, their mean Merle d’Aubigné score increased to 15.42 with excellent, good, fair, and poor rates of 39.2 % (16/41), 46.3 % (19/41), 14.6 % (6/41), and 0 % (0), respectively (the result was based on the last follow-up for each patient). Their mean Harris score increased to 92.3.

## Discussion

To date, scholars have not found an efficient and systemic treatment method in the treatment of hip infections. Once such infections occur, they will cause serious hip joint dysfunction or even lifelong disability even after healing. The previously adopted implantation of antibiotic-loaded cement beads and nonfunctional spacers into an infected hip can neither meet the demands for patients’ walking and hip functional protection during infection treatment period, nor bring about a satisfactory recovery rate [12]. Even worse, the implantation may lead to hip joint hypofunction after second-stage revision hip arthroplasty due to muscular contracture and bone mass loss around the hip [13].

In the present study, a temporarily functional antibiotic-loaded cement prosthesis was implanted after first-stage debridement. This prosthesis can prolong the release time of vancomycin in a great deal to form a long-term effective bactericidal concentration in local tissues, which ensures complete infection healing. The recovery rate in this study was 100 %, and no recurrent infection was found according to the 37 months’ follow-ups. The adoption of vancomycin in this study was mainly based on its role as the first-line antibiotic in the treatment of joint infections: It is resistant to high temperature when mixed and solidified with bone cement, is released slowly in local areas for long time, and has a good curative effect on infections [14]. Vancomycin has the following virtues: It can reach a peak concentration rapidly in a local area to effectively kill bacteria and to reduce the production of drug resistance bacteria; its direct administration to a lesion site can avoid drug effect reduction due to insufficient blood supply caused by infection; further, only a small part of it can enter into the whole body blood circulation, which only results in slight toxic effects on important organs [15]. More than 50 % of the pathogenic bacteria responsible for post-THR infections are gram-positive cocci, on which vancomycin has the best effect among different antibiotics [16]. Bone cement mixed with 5 % vancomycin does not have obviously reduced mechanical strength as compared to cement alone, which, thus, can guarantee weight-bearing walking during treatment. On the other hand, the loaded vancomycin can be released gradually around the hip joint, performing a good long-term bacterium-killing effect. The present study did not show that vancomycin caused any damage to liver and kidney function.

The placement of a rigid framework into the temporarily functional prosthesis can ensure that the prosthesis possesses sufficient weight-bearing strength. This placement enables patients to walk with the aid of crutches, effectively protects the muscles and soft tissues around the hip joint and maintains good tensions of and blood supplies for the muscular tissues around the hip. Meanwhile, it prevents bone loss due to prolonged bed rest and enhances the anti-infection capacity of local tissues. In the present study, the mean Harris score was 46.7 before debridement, which increased to 66.5 during the operative interval and then to 92.3 after the second operation. Both of these scores were better than that reported in another study involving the application of prostalac system [17]. Patients in this study could walk during the whole treatment period, and their life quality was greatly improved. No prosthetic fracture occurred during the treatment period. In addition, although antibiotic-loaded cement prosthesis on the acetabular side was not used in this study, the results did not show walking pain among the patients or a reduced infection recovery rate of them.

The infected hip treatment system in this study is made using a patent die (Chinese utility patent no.: 1072294) and 5 % vancomycin-containing bone cement. The whole making process is simple and highly repeatable. A proper prosthesis can be made according to the sizes of the femoral marrow cavity and acetabulum during operation. Compared to the prostalac system [18], this system is simpler in use and much lower in treatment costs, and meanwhile better improves patients’ life quality, brings about a higher Harris score, and increases infection recovery rate. In this study, the interval between the first and second operations was prolonged to 12–24 weeks rather than the conventional 6–12 weeks. This prolongation was out of the following considerations: (1) There is still an infection recurrence rate between 10 and 15.1 % after one- or two-stage antibiotic-containing revision hip arthroplasty (6); (2) 5 % vancomycin in bone cement is still at an effective bacterium-killing concentration in local areas at 24 weeks; and; (3) antibiotics in temporary prosthesis can work for more than 4 months in vivo [19].

The consequences of infected hips are disastrous, and their treatment has become the difficulty in joint surgery. Although the successful application of prostalac system has given great encouragement and inspiration to scholars, its complicated manufacturing process and high treatment costs force them to develop systems which are simpler in use, lower in cost, better in hip functional protection, and higher in infection recovery rate. In the present study, we used self-developed temporarily functional antibiotic-containing cement prosthesis, whose clinical value for infected hips is initially confirmed.
